# Automated GenePy gene-burden computation via a reproducible Nextflow workflow integrated with the Genomics England (GEL) Lifebit platform

**DOI:** 10.1093/nargab/lqag113

**Published:** 2026-09-28

**Authors:** Iman Nazari, Guo Cheng, James Ashton, Sarah Ennis

**Affiliations:** Department of Human Genetics and Genomic Medicine, University of Southampton, Southampton SO16 6YD, United Kingdom; Department of Human Genetics and Genomic Medicine, University of Southampton, Southampton SO16 6YD, United Kingdom; National Institute for Health Research Southampton Biomedical Research Centre, Southampton SO16 6YD, United Kingdom; Department of Human Genetics and Genomic Medicine, University of Southampton, Southampton SO16 6YD, United Kingdom; Department of Paediatric Gastroenterology, Southampton Children’s Hospital, SouthamptonSO16 6YD,United Kingdom; Department of Human Genetics and Genomic Medicine, University of Southampton, Southampton SO16 6YD, United Kingdom; National Institute for Health Research Southampton Biomedical Research Centre, Southampton SO16 6YD, United Kingdom; Department of Human Genetics and Genomic Medicine, University of Southampton, SouthamptonSO16 6YD,United Kingdom

## Abstract

Interpretation of rare-disease genomes remains constrained by variant-centric analytical frameworks that insufficiently capture the cumulative impact of multiple variants within a gene. GenePy provides an individual-level, gene-based burden metric that integrates variant consequence, allele frequency, and zygosity into a unified quantitative score, enabling a transition from discrete variant annotation to aggregated gene-level interpretation. In the context of Genomics England (GEL), this formulation supports a panel-agnostic, genotype-to-phenotype diagnostic strategy for unresolved monogenic disorders by prioritizing genes with elevated mutational burden per individual. Here, we present a fully automated, containerized GenePy workflow deployed through Nextflow and integrated within the GEL Research Environment via the Lifebit CloudOS platform. This implementation provides scalable, secure, and governance-compliant computation of gene-level burden scores across population-scale cohorts. The workflow harmonizes variant annotation, quality control, and chunked data aggregation within modular, reproducible processes designed for high-throughput execution on cloud-native infrastructure. By enabling robust, portable, and auditable gene-level scoring across large rare-disease sequencing datasets, this framework enhances analytical resolution and supports downstream statistical prioritization, integrative phenotype matching, and hypothesis generation within genotype-to-phenotype diagnostic workflows.

## Introduction

GenePy [1] is a computational framework that converts next-generation sequencing (NGS) data into individual-level gene-burden scores by integrating key variant attributes, including predicted functional consequence, allele frequency, and zygosity, into a unified metric. This formulation shifts the interpretive focus from isolated variant calls towards a more biologically coherent quantification of cumulative gene perturbation. Such gene-level representation is particularly advantageous for investigating complex diseases, in which pathogenicity typically reflects the aggregate contribution of multiple moderate-impact variants rather than singular high-effect alleles. Initial applications of GenePy have demonstrated its capacity to enhance detection of gene–disease associations, support pathway-level analyses, and integrate seamlessly with multiple deleteriousness metrics [2]. Accordingly, GenePy holds great potential for gene discovery by enabling systematic prioritization of candidate genes for downstream validation.

The Genomics England (GEL) Research Environment [3] provides a unique platform for deploying such frameworks because of its secure, accredited architecture and access to the National Genomic Research Library, which includes large-scale whole-genome and whole-exome sequencing data linked to phenotype-rich clinical records. The strict governance model of the GEL environment, which restricts identifiable data to a secure workspace while supporting reproducible computation, necessitates the use of portable, containerized workflows that maintain consistent behaviour across heterogeneous computing environments. Nextflow [4] offers an ideal solution for orchestrating GenePy, enabling modular, version-controlled workflow design with explicit management of dependencies and parallelization. By packaging all components in Docker [5] or Singularity [6] containers, the workflow achieves complete reproducibility, transparent provenance, and seamless deployment across cloud, high-performance computing (HPC), and restricted-access environments. When executed through the Lifebit CloudOS platform [7], which leverages Amazon Web Services (AWS) [8], the pipeline benefits from elastic scaling, managed scheduling, standardized metadata capture, and full compliance with GEL data-governance requirements.

By integrating GenePy within a cloud-native, containerized Nextflow framework, this work provides a scalable, reproducible, and governance-compliant solution for computing gene-level perturbation scores across population-scale genomic datasets, enabling deeper interrogation of complex trait architecture and reproducible integration with statistical and machine-learning analyses (Fig. 1). We emphasize that the algorithmic contribution of this work is as previously described with respect to the underlying GenePy scoring methodology [1,2]. Rather, the primary contribution of this manuscript is the engineering effort required to make GenePy operable within the Genomics England Research Environment. Specifically, this includes the containerization of the pipeline, the development of CloudOS-compatible resource profiles, and the automated orchestration of the multi-step GenePy workflow. These components collectively enable GenePy to run reliably within GEL’s secure, access-controlled infrastructure, where conventional software installation and data movement are not possible. Beyond the specific gene-burden analyses presented here, this infrastructure integration also enables broader downstream applications of GenePy within the GEL environment, including gene prioritization and novel gene discovery, thereby extending the utility of GenePy as a reusable analytical resource within a secure, governance-compliant research infrastructure. The full workflow, including source code, container definitions, and configuration files, is openly available at the GitHub repository UoS-HGIG/Genepy_GEL_V2 and Zenodo https://doi.org/10.5281/zenodo.20527466.

**Figure 1.**
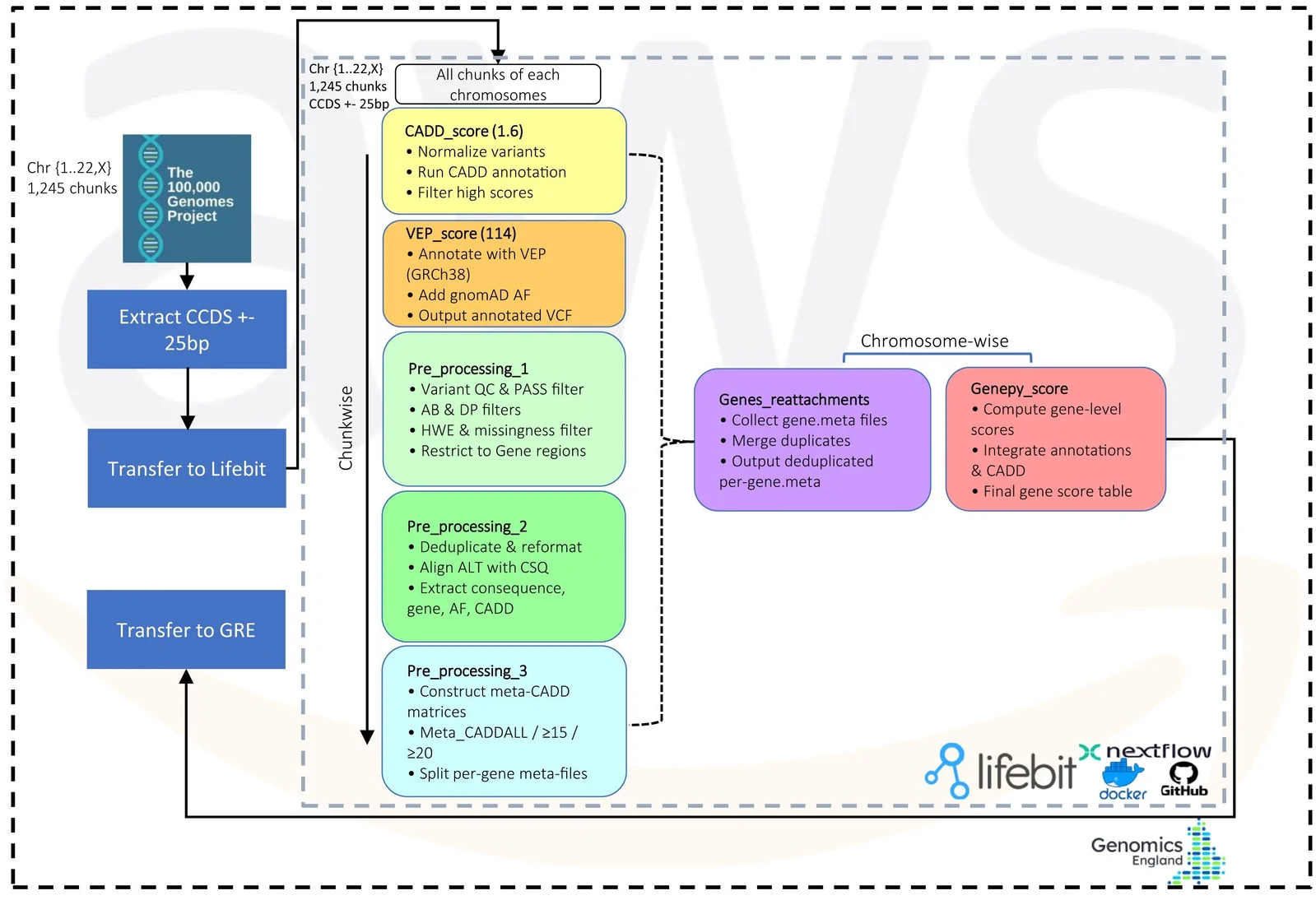
A scalable Nextflow-orchestrated workflow integrating Lifebit CloudOS and GEL infrastructure to compute GenePy gene-level pathogenicity scores, beginning with CCDS *± 25* bp extraction from 100kGP data and progressing through CADD and VEP annotation, multi-stage variant preprocessing, gene-level re-aggregation, and final score generation.

## Methods

### Data sources and processing environment

All analyses were performed within the Genomics England Research Environment using aggregated germline whole-genome sequencing (WGS) data from ~78 000 participants in the 100 000 Genomes Project (AGG2 release). Raw input variant data were provided as per-chromosome VCF chunks (~80–200 GB per chunk) and were integrated with participant-level metadata available within the secure environment. To reduce computational and storage burden while retaining regions of primary clinical interpretability, variants were subset to CCDS coding regions plus *± 25* bp flanking sequence. This extraction step was executed inside the Genomics England Research Environment, producing intermediate per-chromosome files of ~1–6 GB on average.

Computation was then conducted through the Lifebit CloudOS platform, which provides AWS-based elastic compute resources under Genomics England governance controls, after mounting the extracted CCDS *± 25* bp dataset from the Genomics England environment into Lifebit for workflow execution. Pipeline outputs were formatted as a user-friendly matrix of gene-level pathogenicity scores per participant, enabling computationally efficient downstream analyses compared with repeated access to full WGS-scale files. This design emphasizes scalability through cloud execution and automation while maintaining data governance appropriate for sensitive large-scale genomic data, transforming multi-terabyte WGS inputs into a compact analysis-ready representation.

### Workflow design and orchestration

The GenePy workflow was implemented using Nextflow DSL2. The design comprises modular, containerized processes for variant preprocessing, annotation, harmonization, and gene-level scoring. Workflow modules were version-controlled and executed within fixed Docker/Singularity images to ensure deterministic behaviour. Nextflow’s execution engine handled resource allocation, parallelization of genomic chunks, and provenance capture, including command logs, container digests, and input checksums.

### Variant normalization and multisource annotation

Per-chromosome VCFs were partitioned into genomic windows and processed in parallel. Variants were to a canonical representation and annotated using Ensembl VEP (v114) [9] with GRCh38 gene models and gnomAD [10] population allele frequencies. Predicted deleteriousness was incorporated using CADD v1.6 [11].

### Quality control and harmonization

A multi-criteria quality-control framework was applied [12,13], including genotype filtering based on depth and allele balance, removal of loci failing caller-specific filters, Hardy–Weinberg equilibrium assessment [14], and missingness evaluation. Harmonization steps resolved multiallelic representation, ensured one-to-one alignment between sequence variants and VEP annotations, and merged overlapping chunk boundaries into unified variant records. To account for sex-specific ploidy on the X chromosome, genotypes were normalized in a karyotype-aware manner so that downstream analyses could treat chrX genotypes consistently across individuals. In particular, haploid calls in males on chrX were recoded to an equivalent diploid representation to preserve the intended allele dosage and zygosity interpretation for gene-level burden scoring.

### Gene-level data structuring

Variant annotations were aggregated into compact gene-level metadata files encoding coordinates, allele states, consequences, deleteriousness scores, allele frequencies, and QC flags. A genome-wide re-aggregation module merged per-chunk outputs into a single canonical record per gene, harmonizing identifiers across Ensembl and HGNC nomenclature.

### GenePy scoring

Gene-level perturbation scores were generated using the GenePy scoring module, which rescales CADD scores, integrates allele frequencies, encodes genotype states, and computes aggregated severity-weighted burden measures for each individual. The resulting gene *×* sample score matrix provides a quantitative substrate for statistical association testing, enrichment analysis, and machine-learning applications.

### Execution summary and outputs

The workflow was executed in a chunk-wise parallelized manner on Lifebit CloudOS (AWS backend), a managed cloud platform optimized for scalable Nextflow execution, with automatic retries governed by Nextflow’s error-handling framework. The primary output is a comprehensive gene-score table (GenePy matrix); optional outputs include (1) annotated, quality-controlled VCFs for each chunk and (2) per-gene .gene.meta files. All generated artefacts were tracked via checksums and are fully reproducible from the committed workflow configuration and associated Docker images. AWS instance types and their associated hourly costs are detailed in Table 1, and per-chromosome runtime and total cost for a full genome-wide run are reported in Table 2.

**Table 1.** AWS instance types used in this workflow, with hourly cost by region

Instance type	CPU	RAM (GB)	Cost/hour ($)
*eu-west-2 (London)*			
c5.2xlarge	8	16	$0.404
c5.9xlarge	36	72	$1.818
r5.24xlarge	96	192	$7.104
*eu-west-1 (Ireland)*			
c5.2xlarge	8	16	$0.384
c5.9xlarge	36	72	$1.728
r5.24xlarge	96	192	$6.768

**Table 2.** Per-chromosome runtime and total cost for a full genome-wide run, based on eu-west-1 (Ireland) server pricing

Chromosome	Run time	Total cost
Chr 1	15 h 5 min	$494.02
Chr 2	12 h 42 min	$341.36
Chr 3	10 h 3min	$282.32
Chr 4	6 h 18 min	$188.80
Chr 5	17 h 16 min	$412.85
Chr 6	10 h 58 min	$249.09
Chr 7	10 h 58 min	$236.90
Chr 8	14 h 5 min	$231.84
Chr 9	9 h 11 min	$223.28
Chr 10	2 h 5 min	$42.40
Chr 11	11 h 40 min	$298.30
Chr 12	9 h 00 min	$252.36
Chr 13	3 h 9 min	$81.23
Chr 14	7 h 56 min	$181.80
Chr 15	8 h 5 min	$201.88
Chr 16	10 h 32 min	$208.69
Chr 17	12 h 17 min	$300.60
Chr 18	2 h 48 min	$68.98
Chr 19	12 h 58 min	$394.56
Chr 20	8 h 2 min	$147.38
Chr 21	5 h 17 min	$100.67
Chr 22	6 h 27 min	$140.55
Chr X	7 h 9 min	$163.44
**All chromosomes**	**214 h 1 min**	**$5243.28**

We note that the workflow consists of several sequential steps and is executed chunk-wise per chromosome, with each chunk drawing on different computational resources at different stages of the pipeline. Extracting and attributing cost at the level of individual resources within each chunk is technically difficult and would generate an impractically large volume of granular cost data, offering limited additional interpretability. We therefore report cost and runtime aggregated at the chromosome level, which we believe provides the clearest and most practically useful summary of the workflow’s computational performance for prospective users.

### Portability outside the Genomics England environment

The workflow presented in this manuscript has been specifically optimized for execution within the GEL/Lifebit CloudOS environment, and several components, including the resource profiles, container registry access, and executor configuration, are tailored to this platform. As such, this particular implementation is not directly portable to a local HPC cluster without modification. However, we have developed a separate, related Nextflow implementation of GenePy specifically designed to run on local HPC infrastructure using an SLURM executor (UoS-HGIG/GenePy-2), available at https://github.com/UoS-HGIG/GenePy-2. We consider this separation of platform-specific implementations to be a deliberate design choice, allowing each version to be optimized for its intended computational environment rather than compromising performance or maintainability with a single, more generic but less efficient codebase.

### Data and code availability for reproducibility

Due to the sensitive nature of the data within the Genomics England Research Environment, patient-identifiable information cannot leave the environment, and consequently the original input data or a derived test dataset cannot be provided within the public GitHub repository or Zenodo archive. This is a necessary constraint of working within GEL’s secure, access-controlled infrastructure rather than a limitation of the workflow itself. To support reproducibility within this constraint, the file path to an original input chunk within the GEL Research Environment, along with the corresponding output results, is available upon request to approved GEL users wishing to independently verify successful installation and execution of the pipeline prior to running it across the whole genome.

## Results

### GenePy score analysis reveals gene-level pathogenic burden in a panel-based rare-disease cohort

To assess the aggregate pathogenic burden across our cohort, we applied GenePy scoring, a gene-level pathogenicity metric that integrates variant-level deleteriousness predictions, population allele frequencies, and individual zygosity, to ~24 000 unique genes in the human reference genome across 78,000 individuals. GenePy transforms variant-level interpretation into an intuitive per-gene, per-individual score, enabling systematic comparison of genetic burden both across individuals and between case and control populations.

To examine gene-specific pathogenic-variant distributions across the cohort, we visualized GenePy density plots for three genes representing distinct disease mechanisms (Fig. 2). SERPINF1 exhibited the most complex distribution, with multiple density peaks indicating heterogeneous variant burden across the cohort, potentially reflecting both founder effects and recurrent pathogenic alleles in specific subpopulations. PRPF3 displayed a broader distribution with a secondary mode, reflecting a modest subset of individuals carrying rare deleterious variants. In contrast, REEP6 showed a sharp peak near zero with minimal right-tail distribution, suggesting extremely low population-level pathogenic burden and high constraint. These density profiles provide a cohort-wide perspective on the penetrance and frequency of pathogenic variation within clinically actionable genes, facilitating prioritization for downstream functional validation and gene–phenotype association studies.

**Figure 2.**
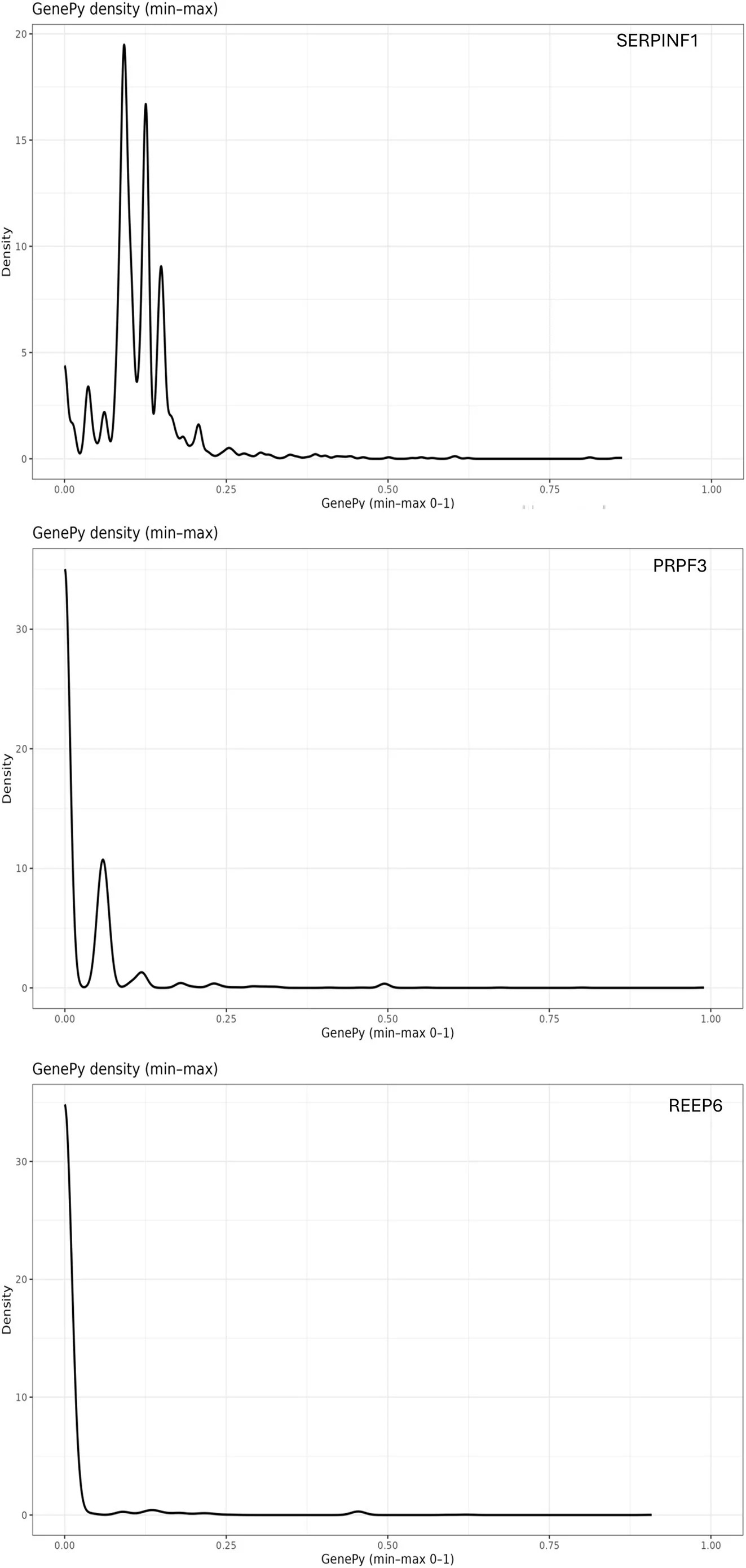
Cohort-wide GenePy score density plots, shown top to bottom for SERPINF1, PRPF3, and REEP6.

To validate the discriminatory power of GenePy in a phenotype-specific context, we compared the distribution of GenePy scores for three diagnostically relevant genes (COL1A1, KCNJ1, and APRT) between cases presenting with the corresponding phenotypes (Osteogenesis imperfecta for COL1A1; nephrocalcinosis/nephrolithiasis for KCNJ1 and APRT) and control individuals from the broader cohort without these diagnoses (Fig. 3). All three genes demonstrated a striking bimodal distribution, with the vast majority of both case and control individuals clustering at GenePy scores near zero, reflecting the rarity of pathogenic variants in the general population. However, cases exhibited a distinct secondary peak at higher GenePy scores (inset panels), representing individuals harbouring deleterious compound-heterozygous or homozygous variants.

**Figure 3.**
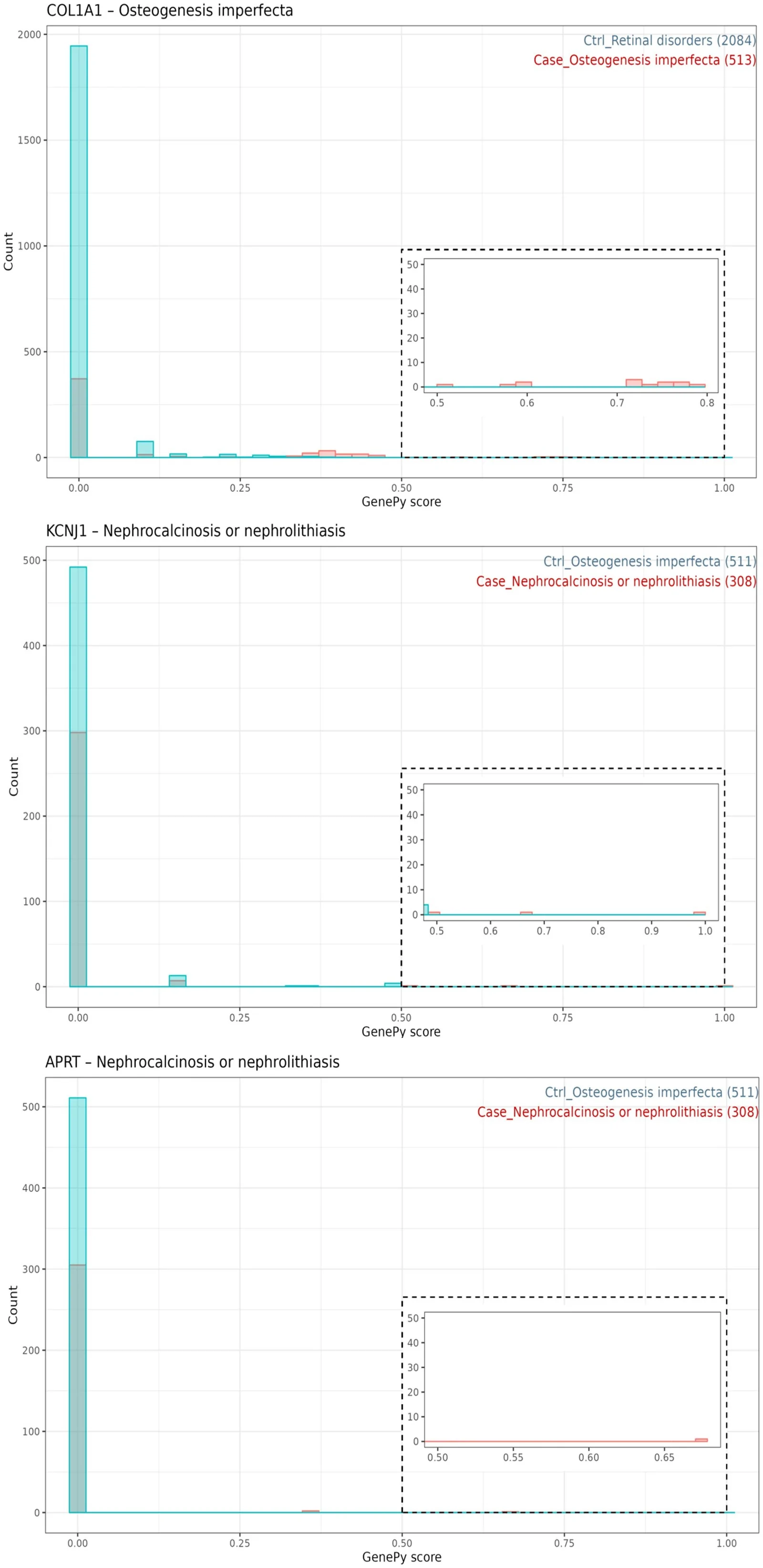
Case–control GenePy score distributions, shown top to bottom for COL1A1, KCNJ1, and APRT.

The genome-wide Manhattan-style plots in Fig. 4 illustrate the distribution of GenePy scores across all chromosomes for three representative patients, revealing elevated pathogenic scores in genes previously implicated in their respective phenotypes. Notably, DNM2 (limb-girdle muscular dystrophy), MFSD8 (hereditary ataxia), and DNAH5 (associated with primary ciliary dyskinesia) exhibited markedly elevated GenePy scores in affected individuals compared with the baseline genomic background, consistent with their clinical diagnoses obtained through structured phenotypic questionnaires.

**Figure 4.**
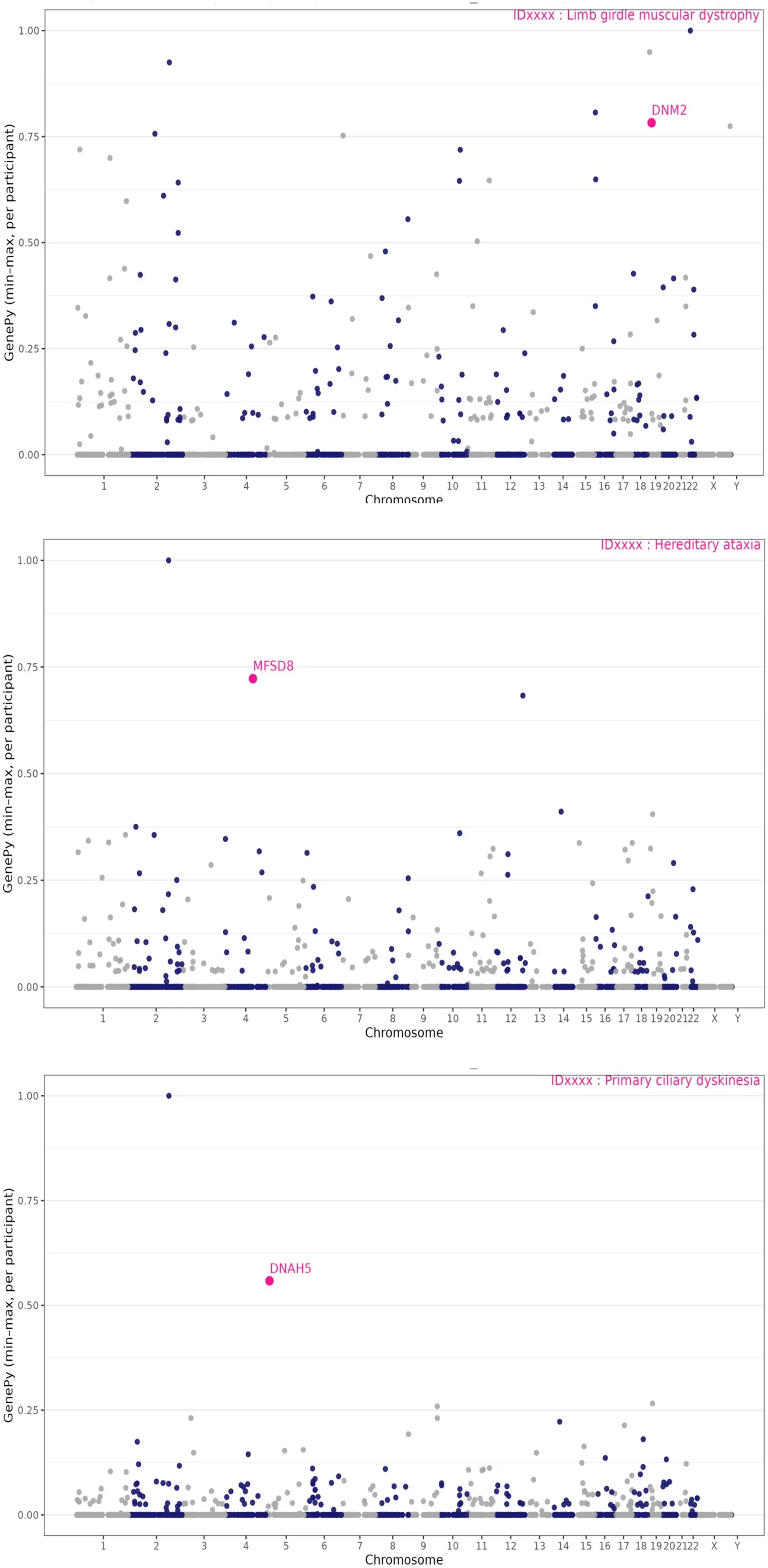
Manhattan-style genome-wide GenePy plots for three representative patients, highlighting elevated scores in phenotype-relevant genes.

## Discussion

The implementation of GenePy within the Genomics England Research Environment enabled robust, scalable computation of gene-level burden metrics across whole-genome sequencing datasets. However, financial constraints associated with cloud-based computation necessitated a targeted analytical strategy for the AGG2 application reported here, such that cost limitations related to AWS compute utilization precluded comprehensive genome-wide processing of all annotated loci within the available funding. Accordingly, as described in the Methods, analyses were restricted to CCDS regions with an additional *± 25* bp flanking window, an interval-focused design that captures canonical coding bases and immediate splice-adjacent sites while maintaining computational affordability in the cloud-governed Lifebit execution environment.

While narrower in scope than whole-genome interrogation, this strategy retained sensitivity to variants with established functional relevance and substantially reduced computational overhead. The CCDS *± 25* bp targeting should therefore be considered a configurable implementation choice rather than a fixed limitation of the pipeline. In future releases (for example, AGG3), the workflow could be extended to broader, gene-centric intervals, including whole gene bodies (coding and non-coding sequence) and expanded flanking regions spanning promoter through 5$^{\prime }$ UTR to 3$^{\prime }$ UTR, where inclusion of regulatory context (for example, promoters and enhancers) is desirable. Beyond single-locus intervals, the same scoring paradigm could be extended to digenic or oligogenic models by aggregating gene-level scores across biologically or phenotypically related gene sets to prioritize multi-gene hypotheses for follow-up.

Despite the restricted genomic window, this framework provided meaningful insights into the broader landscape of genetic perturbation. GenePy inherently integrates distributed variant effects into a unified, gene-level measure, enabling quantification of cumulative pathogenic burden even when variant contributions arise from modest-effect alleles. Importantly, the use of a gene-centric severity score allows signals originating from non-coding yet functionally relevant bases, such as splice-region variants, regulatory motifs adjacent to exons, or elements influencing transcript stability, to be partially captured within the defined CCDS *± 25* bp interval. While the window does not encompass distal enhancers, promoters, or long-range regulatory architecture, the scoring framework nonetheless improves interpretability of near-coding variation, which frequently contributes to disease mechanisms but can be overlooked in variant-centric analyses. Because GenePy is driven by variant annotations and deleteriousness metrics, its performance in near-coding and other regulatory-proximal regions should improve as these annotations and predictors improve.

More broadly, the successful deployment of this workflow demonstrates how cloud-native, containerized pipelines can support scalable, governance-compliant analysis of population-scale genomic datasets. The Nextflow–Lifebit architecture ensured reproducibility, provenance tracking, and fault-tolerant execution while conforming to GEL’s secure operational model. Even under financially constrained computational budgets, the workflow provided consistent and interpretable gene-level burden estimates suitable for downstream statistical modelling and machine-learning applications. Expanding future iterations of the workflow to encompass whole-genome non-coding regions, including enhancers, promoters, and regulatory domains, will be an important step towards fully characterizing polygenic architecture and understanding the distributed pathogenicity landscape underlying complex traits. Complementing this will be the integration of additional deleteriousness metrics and functional priors tailored to non-coding annotations, which are expected to further enhance the interpretive power of gene-based burdens across the genome.

## Data Availability

By integrating GenePy within a cloud-native, containerized Nextflow framework, this work provides a scalable, reproducible, and governance-compliant solution for computing gene-level perturbation scores across population-scale genomic datasets, enabling deeper interrogation of complex trait architecture and reproducible integration with statistical and machine-learning analyses (Figure 1). The full workflow, including source code, container definitions, and configuration files, is openly available at the GitHub repository https://github.com/UoS-HGIG/GenePy_GEL_V2 and Zenodo https://doi.org/10.5281/zenodo.20527466.
